# Heterogeneity Among Neutrophils

**DOI:** 10.1007/s00005-017-0476-4

**Published:** 2017-05-30

**Authors:** Marzena Garley, Ewa Jabłońska

**Affiliations:** Department of Immunology, Medical University of Bialystok, J. Waszyngtona 15A, 15-269 Białystok, Poland

**Keywords:** Neutrophils, PMNs, Heterogeneity, Subpopulations, Populations

## Abstract

Neutrophils (PMNs) play a key role in innate defence mechanisms. Generally, PMNs were considered to have a homogeneous population of mature and diversified cells. It seems, however, that their pleiotropic action results from the existence of different subpopulations in this group of cells. There are data that confirm the involvement of PMNs in the direct activation of other cells in non-specific response, as well as specialised cells in specific response. For example, there have been observations of PMNs with different levels of activity in relation to lymphocytes, and a population was identified which had characteristics similar to those of cells which are capable of presenting antigens. There are also reports of PMNs which demonstrate different survival time or capacity for chemotaxis. Other studies suggest that the neutrophil response to *Staphylococcus aureus* is diverse (not identical among all neutrophil). There are also reports of PMNs with varying activity during inflammation, which might explain many as yet unknown pathophysiological aspects of their hyperreactivity. The functional dualism of PMNs in the course of neoplastic disorders raises a lot of controversy. This paper presents the current state of knowledge of the heterogeneity of PMNs and their potential roles in different stages of disease.

## Introduction

Neutrophils (PMNs: polymorphonuclear cells) for a long time were considered as fully differentiated effector cells of the inflammatory response. It was believed that their fundamental role was extracellular phagocytosis involving reactive oxygen species (ROS), cationic proteins and enzymes. In recent years, numerous studies have shown a wider range of functions these cells possess. Newly discovered effector molecule repertoire of neutrophils includes an array of cytokines, neutrophil extracellular traps and other molecules of innate humoral immunity. Neutrophils are involved in the activation and regulation of effector functions in other cells of the non-specific response, as well as of cells of specific immunity. It was confirmed that neutrophils play a key role in the pathogenesis of numerous disorder, from extra- and intracellular infections, such as chronic inflammation, autoimmunization or neoplastic disorders (Mantovani et al. 2011).

In light of this information, a question arises: do neutrophils form a heterogeneous cell population? Discoveries of new functions of leukocytes: lymphocytes, monocytes, were accompanied by indicating a new, specific population of cells with a characteristic antigen coat. Considering the wide range of neutrophil activities, it seems reasonable to look for subpopulations of these cells.

## First Reports on Neutrophil Subpopulations

The search for neutrophil heterogeneity started in 1970–1980s. At the time research was focused on evaluating the functional differences of neutrophils, their density, and the biosynthesis of proteins/RNA (ribonucleic acid) (Broxmeyer et al. 1980; Harvath and Leonard 1982; Klempner and Gallin 1978; Pember et al. 1983; Ramsey 1972; Seligmann et al. 1981). It was found that different groups of these cells vary in their capacity for chemotaxis (Ramsey 1972). Also, neutrophils were shown to demonstrate varied myeloperoxidase (MPO) activity (Pember and Kinkade 1983). In our research, we identified two populations of neutrophils with different expression of MPO (MPO^low^ and MPO^high^) in the presence of inflammatory cytokines. Moreover, stimulating neutrophils with lipopolysaccharide and fMLP (*N*-formylmethionyl-leucyl-phenylalanine) was shown to separate two populations of cells which differed in MPO expression as well as size (to be published). The use of monoclonal antibodies made it possible to confirm the hypothesis about the existence of subpopulations of neutrophil granulocytes. This was shown by detecting a 5% population of neutrophils without the CALLA/CD10 antigen (common acute lymphoblastic leukaemia antigen) (Clement et al. 1983; McCormack et al. 1987). Other studies found that intravenous administration of endotoxins recruits only a “specific” population of blood marrow neutrophils (Brown et al. 1989). A little later, a hyperreactive subpopulation of neutrophils was identified in patients with acute respiratory failure (Chollet-Martin et al. 1992).

Nevertheless, the state of knowledge at the time, as well as technical possibilities made it impossible to confirm the importance of neutrophil subpopulations, as seen in a meaningful article published by Gallin (1984) in Blood, “Neutrophil heterogeneity exists, but is it biologically meaningful?”. Years of research were devoted to neutrophils yet the questions still remains valid.

The International Society of Blood Transfusion Granulocyte Antigen Working Party in 1998 established a nomenclature of granulocyte antigens consisting of five antigen systems. Human neutrophil antigens (HNAs) comprise a group of glycoproteins expressed on human neutrophils: HNA-1 (FcγRIIIb, CD16), HNA-2 (CD177), HNA-3 (CTL2), HNA-4 (CD11b/CD18; Mac-1, CR3), HNA-5 (CD11a/CD18) (Bux 1999) (Table 1).

**Table 1:** Neutrophil subsets

Neutrophil subset	Immunophenotype	Functional properties	References
Mature/classic	HNA-1 (FcγRIIIb, CD16) HNA-2 (CD177) HNA-3 (CTL2) HNA-4 (CD11b/CD18; Mac-1, CR3) HNA-5 (CD11a/CD18)		Bux (1999)
Long-living	HLA-DR, CD80, CD49d	IL-8, IL-1Ra, IL-1-β	Chakravarti et al. (2009)
Aged	CD62L^low^CXCR4^high^CD11B^high^CD49^high^	Phagocytosis, NETosis	Casanova-Acebes et al. (2013), Rankin (2010), Zhang et al. (2015)
N_BH_		BAFF, APRIL	Puga et al. (2011)
TCR^+^	TCRαβ	IL-8 inhibition of apoptosis	Puellmann et al. (2006)
PMN-I	TLR2/TLR4/TLR5/TLR8 CD49d^+^CD11b^–^	IL-12, CCL3	Tsuda et al. (2004)
PMN-II	TLR2/TLR4/TLR7/TLR9 CD49d^–^CD11b^+^	IL-10, CCL2	
PMN-N	TLR2/TLR4/TLR9 CD49d^−^CD11b^−^		
mPR3^+^	mPR3^high^	Outbreak or progression of chronic inflammatory	Witko-Sarsat et al. (1999)
	mPR3^+^CD177^+^	Wegener’s granulomatosis	Bauer et al. (2007)
CD177	High, low, negative expression		Wu et al. (2016)
LDNs	CD66b, CD11b^+^CD16^+^ and/or CD11b^low/–^CD16^low/–^		Deng et al. (2016), Giallongo et al. (2015), Grayson et al. (2015), Hossain et al. (2015), Jiang et al. (2014), Liu et al. (2010), Mare et al. (2015), Marini et al. (2016), Midgley and Beresford (2016)
LDNs/G-MDSCs	CD66b^+^CD15^+^CD14^–/dim^CD33^dim^HLA-DR^−^	Immunosuppressive	Jiang et al. (2014), Mandruzzato et al. (2016), Solito et al. (2014)
LDGs	CD15^+^/CD14^low^, CD10^+^/CD14^low^, CD16^high^/CD86^−^	IFN-γ, TNF-α pro-inflammatory	Denny et al. (2010)
Bone marrow-derived immature neutrophils	CD10^low/−^CD16^low^		Manz and Boettcher (2014)
MDSCs	CD11c^bright^/CD62L^dim^/CD11b^bright^/CD16^bright^	Inhibition of T lymphocytes proliferation	Pillay et al. (2012)
MDSCs in cancer	CD14^+^ CD33^+^ HLA-DR^−^	Inhibition of T lymphocytes function	Almand et al. (2001), Hoechst et al. (2008), Kusmartsev et al. (2008), Rodriguez et al. (2009)
N1	CD11b^+^/Ly6G^+^	Anti-neoplastic activity CCL-3, CXCL9, CXCL10 IL-12, TNF-α, GM-CSF	Fridlender et al. (2009), Fridlender and Albelda (2012), Pekarek et al. (1995), Shen et al. (2007)
N2		Pro-neoplastic properties VEGF, MMP-9	Jabłońska et al. (2012), Schmielau and Finn (2001), Tazawa et al. (2003)
Proangiogenic	CXCR4^high^/VEGFR1^high^	MMP-9	Christoffersson et al. (2012)

*IL-1Ra* interleukin-1 receptor antagonist, *N*_*BH*_ B-cell helper neutrophils, *BAFF* B-cell activating factor, *APRIL* a proliferation-inducing ligand, *NETs* neutrophil extracellular traps

## Neutrophil Populations with Different Survival Time

Survival of neutrophils has recently been the point of numerous scientific disputes. It is commonly agreed that human PMNs survive in blood for up to 8 h and then transfer into tissues, where they live for 1–2 days. Most recent reports indicate that neutrophil survival time may be significantly longer, lasting up to 90 h (Pillay et al. 2010). Longer lifespan of neutrophils may set the basis for PMNs to undergo phenotypic and functional changes and account for neutrophil heterogeneity (Silvestre-Roig et al. 2016).

It was found that there are subpopulations of human PMNs with a characteristic phenotype, showing the expression of HLA-DR (human leukocyte antigen DR), CD80, and CD49d molecules, which are characterised by a significantly extended survival of up to 72 h. These cells compose 8–17% of non-apoptotic neutrophils which produce significant amounts of superoxide anions and leukotrienes. Long-surviving neutrophils demonstrate an elevated phagocytic index and increased adhesion, as well as a limited capacity for chemotaxis and exocytosis of primary and secondary granules. Research has shown that stimulation of PMNs (isolated from human blood) with granulocyte–macrophage colony-stimulating factor (GM-CSF), tumour necrosis factor (TNF)-α, and interleukin (IL)-4, all of which exist in inflammation sites, leads to generating long-living populations of neutrophils producing significant amounts of IL-8, IL-1 receptor antagonist, and IL-1β. The newly found subpopulation of human neutrophils is characterised by a unique profile of intracellular signalling molecule phosphorylation. Researches demonstrated an involvement of PI3K pathway kinases in extending the survival of identified neutrophil subpopulations. The results of these studies suggest that PMNs are capable of switching from a “classic” phenotype to a “long-living neutrophils” depending on the environmental conditions of the host (Chakravarti et al. 2009).

Under steady-state conditions, neutrophil heterogeneity may arise from ageing and replenishment by bone marrow-released neutrophils. Aged mouse neutrophils upregulate chemokine receptor 4 (CXCR4) and express low levels of l-selectin. The aged subset has hypersegmented nucleus, reduced size and granularity (Casanova-Acebes et al. 2013; Rankin 2010; Zhang et al. 2015).

Hypothesis that different maturity levels of human PMNs contribute to different types of PMNs has not been unequivocally confirmed.

## Neutrophil Heterogeneity in Relation to Immunoregulatory Functions

Neutrophils are an important element of the innate immune system, although recently their role as regulator and effector cells in innate immunity mechanisms is also recognised (Mócsai 2013; Nathan 2006; Németh and Mócsai 2012).

Puga et al. (2011) showed that there is a certain pool of neutrophils called B-cell helper neutrophils, found in the marginal zone of the spleen. These neutrophils manifest a capacity for producing significant amounts of cytokines, for example TNF superfamily proteins, such as B-cell activating factor and a proliferation-inducing ligand, which have a strong effect on B lymphocyte proliferation and production of immunoglobulins (Puga et al. 2011).

Under certain conditions, neutrophils are able to take features of antigen-presenting cells, e.g., cross-presenting ovalbumin. It was observed that these cells absorb and present exogenous antigens, stimulating differentiation into cytotoxic lymphocytes through direct interaction between neutrophils and naive CD8^+^ T cells (Beauvillain et al. 2007; Pelletier et al. 2010).

Also, it was rather surprising to find a T-cell receptor αβ (TCRαβ receptor) on the surface of human neutrophils comprising a 5–8% subpopulation. Research showed that the expression of this receptor is accompanied by RAG1/RAG2 recombinase. It was also found that expression of TCRαβ and the RAG1/RAG2 complex (recombination activating gene 1/2 complex) in vivo is regulated by G-CSF. Moreover, it was demonstrated that stimulation of this immunoreceptor in neutrophils results in increased secretion of IL-8 and inhibition of apoptosis. These findings were confirmed in experiments conducted on mice. The results include this subpopulation of neutrophils into the group of cells which demonstrate the features of innate immune response (Puellmann et al. 2006).

## Neutrophil Population Diversity in Response to *Staphylococcus aureus*

Neutrophils play a key role in antibacterial response, which is confirmed by the high rate of bacterial infections in neutropenia patients. Nevertheless, despite correct or sometimes elevated levels of PMNs, it is possible to observe their dysfunction which may lead to sepsis.

Experimental tests provided evidence for the existence of two subpopulations of murine neutrophils with modified activity against *Staphylococcus aureus* and other, with a changed production of cytokines and chemokines, expression of Toll-like receptors (TLR), and different surface antigens and influence on macrophage activation. It was shown that one of these populations produces IL-12 and chemokine ligand 3 (CCL3; and is called PMN-I), while the other secretes IL-10 and CCL2 (PMN-II), compared to the normal neutrophil population (so-called PMN-N). PMN-I population activates macrophages typically (with the involvement of CCL5 and inducible isoform of nitric oxide synthase) and PMN-II performs it in an alternative manner (with the involvement of CCL17 and a mannose receptor), yet PMN-N do not activate macrophages. Cells from the PMN-I population indicate the expression of TLR2/TLR4/TLR5/TLR8, PMN-II cells indicate the expression of TLR2/TLR4/TLR7/TLR9, and PMN-N cells express TLR2/TLR4/TLR9. Also, PMN-I indicate the expression of surface antigens: CD49d^+^CD11b^–^, PMN-II: CD49d^–^CD11b^+^, PMN-N: CD49d^–^CD11b^–^. PMN-I cells were obtained from animals resistant to methicillin-resistant *S. aureus* (MRSA), while MRSA-sensitive mice were the source of the second group of PMNs. Some authors believe that PMN-Ns obtained from naive mice might differentiate into certain populations of neutrophils under the influence of specific factors, such as infection (Tsuda et al. 2004).

Infection or aseptic inflammation is the main postoperative complications. It was believed that such complications may result from the use of polyethylene components or be caused by the release of ultra-high-molecular-weight polyethylene (UHMWPE) from, for example, orthopaedic prostheses. To confirm this thesis, isolated human neutrophils were incubated in vitro with UHMWPE molecules and then added to *S. aureus* cultures. Surprisingly, the cells responded in different manners. It was shown that over 40% of PMNs which absorbed UHMWPE lost the capacity for bacterial phagocytosis. An inhibited bacterium capture was observed in neutrophils exposed to UHMWPE, but also the cells showed an increased respiratory burst. Almost 30% of the PMNs did not contain any UHMWPE molecules, and most of them were capable of absorbing *S. aureus* cells. These findings indicate, firstly, that there are different subpopulations amongst PMNs and, secondly, they explain the cause of complications related to pyogenic infections associated with biomedical implants (Bernard et al. 2007).

## Neutrophil Populations with Different Activity in Inflammation

Many aspects of the pathophysiology of neutrophil hyperreactivity and its role in immune disorders remain largely unknown. Perhaps identifying and describing the populations of neutrophils associated with inflammation might serve in explaining these issues.

Research conducted by Witko-Sarsat et al. (1999) showed that circulating neutrophils form two populations of cells based on the presence or lack of a membrane expression of proteinase 3 (mPR3). It was found that the percentage of mPR3^+^ differs in healthy subjects. Also, it was observed that a large ratio of mPR3^+^ neutrophils among circulatory leukocytes may be a significant factor of an outbreak or progression of chronic inflammatory disorders. This is indicated by an increased number of mPR3^high^ neutrophils in patients with vasculitis and rheumatoid arthritis (Witko-Sarsat et al. 1999).

Other studies looked for co-expression of mPR3 and CD177 molecules, as an effect of cell activation. The authors found CD177 in the population of mPR3^+^ neutrophils. Experiments showed an analogous increase or decrease of the examined molecules during cell stimulation or in the course of spontaneous apoptosis. According to researchers, the source of mPR3 lies in secondary granules and excretory vesicles, as in the case of CD177. PR3 is an important molecule in the course of Wegener’s granulomatosis, which is characterised by an increased level of mPR3^+^CD177^+^ neutrophil subpopulation (Bauer et al. 2007). CD177, in humans, is exclusively expressed on the surface of PMNs and regulates transmigration across the endothelium (Sachs et al. 2007). CD177 expression is required for surface presentation of PR3, which facilitates the transmigration of CD177^+^ neutrophils (Kuckleburg et al. 2012; von Vietinghoff et al. 2007). Membrane PR3 was identified as an antigen ANCA (anti-neutrophil cytoplasmic antibody)-depended vasculitis (Jennette et al. 1990). The expression of CD177 may vary in PMNs and in different individuals (e.g., high, low, negative expression) (Wu et al. 2016). Implication of CD177^+^ or CD177^–^ populations in ANCA-derived vasculitis or other inflammatory diseases remain unknown (Xie et al. 2015). The absence of CD177 did not affect the migratory capacity of neutrophils but it caused cell death (Sachs et al. 2007; Xie et al. 2015).

## Subpopulations of PMNs in Low Density

Centrifugation of blood in the density gradient allows the isolation of two leukocyte fractions: low density cells—peripheral blood mononuclear cells (PBMCs) and higher density cells—PMNs.

Low-density neutrophils (LDNs) are found to sediment within the PBMCs fraction obtained after density gradient centrifugation of blood from patients with cancer or inflammation (Scapini et al. 2016). LDNs display a neutrophil-like morphology and express CD66b, but can be heterogeneous. During inflammatory condition they can be composed of mixed populations of both CD11b^+^CD16^+^ mature and immature CD11b^low/–^ and/or CD16^low/–^ and their frequency often correlates with disease severity and/or responsiveness to treatment (Deng et al. 2016; Grayson et al. 2015; Hossain et al. 2015; Jiang et al. 2014; Liu et al. 2010; Mare et al. 2015; Midgley and Beresford 2016). Why only a group of normal density mature neutrophils become LDNs upon activation remains unknown.

Immunosuppressive LDNs, known as granulocytic myeloid-derived suppressor cells (G-MDSCs), have been discovered within the PBMCs fraction from patients with solid tumours, hematological malignancies and inflammatory disease conditions (Darcy et al. 2014; Favaloro et al. 2014; Giallongo et al. 2015; Gorgun et al. 2013; Janols et al. 2014; Marini et al. 2016; Rieber et al. 2014). LDNs/G-MDSCs suppress proliferation of T cells and/or interferon (IFN)-γ production and are described as CD66b^+^CD15^+^CD14^–/dim^CD33^dim^HLA-DR^–^ cells (Mandruzzato et al. 2016; Solito et al. 2014). They may also express enhanced/reduced levels of maturation markers (CD11b, CD16, CD124/IL-4R), activation markers [CD66b, CD16, CD11b, CD62L, CD54/ICAM-1 (intercellular adhesion molecule 1), CD63, CD274/PD-L1 (programmed death-ligand 1)], functional markers (arginase 1) or chemokine receptors (CXCR2, CXCR4) (Scapini et al. 2016).

There are also bone marrow-derived immature PMNs, revealed as CD10^low/–^CD16^low^ cells, mobilised during severe systemic infections (Manz and Boettcher 2014). Other tests identified a population of mature human neutrophils with a CD11c^bright^/CD62L^dim^/CD11b^bright^/CD16^bright^ antibody coat as a unique, circulating population of myeloid cells (MDSC) capable of suppressing the proliferation of human T cells. These cells were found in patients with inflammation. Some researchers claim that the mechanism of inhibiting the proliferation of T lymphocytes depends on the expression of Mac-1 integrin (macrophage 1 antigen) and ROS released to the immune synapse between PMNs and T cell, which may be the target of modulation strategies of this phenomenon (Pillay et al. 2012).

The presence of proinflammatory LDNs, called low-density granulocytes (LDGs), was reported in blood from patients with inflammatory conditions (Scapini et al. 2016). Different data presented by Denny et al. (2010) showed that in the mononuclear leukocyte fractions isolated by density gradient centrifugation of blood taken from patients with systemic lupus erythematosus, there is a characteristic proinflammatory neutrophil subpopulation, identified as CD15^+^/CD14^low^, CD10^+^/CD14^low^, CD16^high^/CD86^–^ phenotype cells. These neutrophils release increased amounts of type I interferons and IFN-γ, as well as TNF-α, yet they also demonstrate decreased phagocytosis. Moreover, LDGs induce endothelial cell destruction and interfere with its reconstruction processes (Denny et al. 2010). Perhaps the evaluation of the proinflammatory neutrophil subpopulation fractions would prove useful in diagnosing and monitoring inflammatory diseases.

## Neutrophil Heterogeneity in the Neoplastic Process

In recent years, it has been speculated whether tumor cells are able to influence the maturation and differentiation of myeloid cells and, in consequence, change the course of the immune response. Tumor-implanted mice indicated the presence of immunosuppressive PMNs and monocytes, named MDSC, which were found to inhibit the function of T lymphocytes (Ostrand-Rosenberg and Sinha 2009). It is possible, that MDSCs are produced in the bone under the influence of a substance secreted by the tumor cells.

Researchers looked for similar cells in cancer patients. It was found that activated granulocytes in neoplastic patients exhibit similar features to mice MDSC. Others suggest that populations of CD14^+^ cells might be the equivalent of MDSC. Results of other researchers indicate speculate that the CD33^+^ and HLA-DR-negative markers characterise human MDSC (Almand et al. 2001; Hoechst et al. 2008; Kusmartsev et al. 2008; Rodriguez et al. 2009).

Studies of large groups of head and neck, lung, and urinary tract cancer showed the presence of a large number of MDSC and neutrophils at different maturity stages. These neutrophils demonstrated suppressive properties against T cells, such as inhibition of proliferation and production of IFN-γ. Also, immature granulocytes from the MDSC population possessed reduced capacity for migration and chemotaxis (lack of CXCR1 and CXCR2 receptors), limited function (decreased production of ROS and IL-8), and extended lifespan (Brandau et al. 2011).

**Fig. 1:**
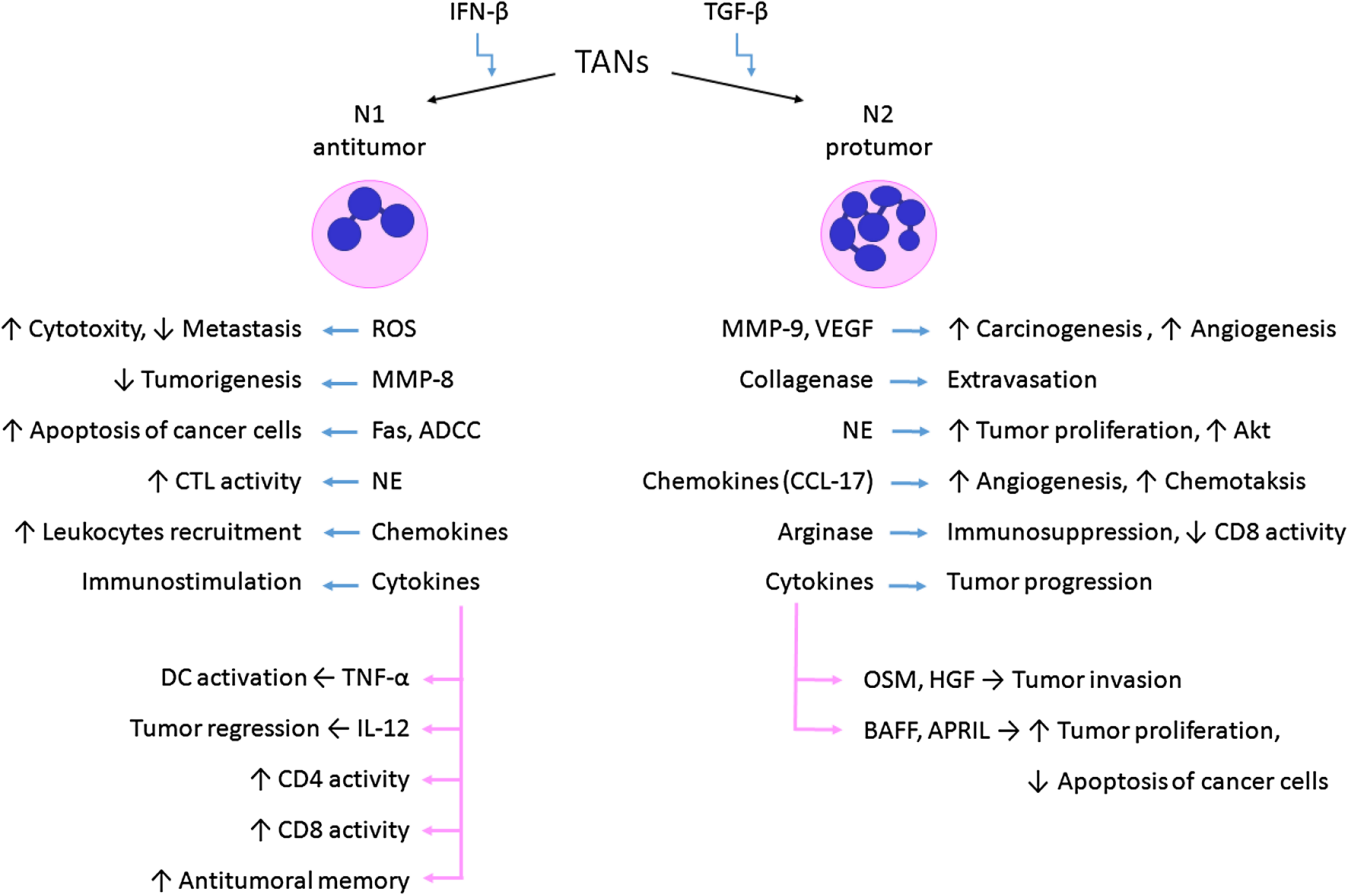
TANs in response to cancer cells. *BAFF* B-cell activating factor, *APRIL* a proliferation-inducing ligand, *OSM* oncostatin M, *HGF* hepatocyte growth factor, *NE* neutrophil elastase, *ADCC* antibody‐dependent cell‐mediated cytotoxicity

Current knowledge about neutrophils suggests that the so-called tumor-associated neutrophils (TANs) and, according to some authors, their precursors (peripheral neutrophils G-MDSC) found in the spleen, bone marrow, and blood play a key role in cancer biology. Still, the results of micro-array studies clearly showed that TANs are a separate neutrophil population from G-MDSC or regular neutrophils (Fridlender and Albelda 2012). However, the role of TANs in response to cancer remains a controversial subject (Fig. 1; Table 2).

**Table 2:** Properties of neutrophils differing neutrophils populations, by Fridlender and Albelda (2012)

	Naive neutrophils	G-MDSC	TANs
Granule proteins			
Primary	+++	+	+
Secondary	+++	Mod	+
Tertiary	+++	Mod	+
Respiratory burst			
Peroxidase	+++	+++	+
Reduced nicotinamide adenine dinucleotide phosphate complex	+++	Mod	+
TLR	+	+++	Mod
Structural genes			
Actin binding	Mod	Mod	+
Cytoskeleton	Mod	Mod	+
Apoptosis			
Intrinsic (BCL2) pathway	Mod	+++ (BH-3)	+
NF-κB–antiapoptotic	+++	+	+
Immune system			
Chemokines	+	Mod	+++
Cytokine activity	+	Mod	+++
APC genes	+	+++	+++

*+++* high, + low, *Mod* moderate

The explanation of the functional dualism of PMNs may be found in the existence of subpopulations of these cells with varying immune properties. It is suggested to divide TANs into N1 and N2. The N1 phenotype is characterised with anti-neoplastic activity, contrary to N2 cells which promote tumor development (Galdiero et al. 2013; Granot and Jablonska 2015; Kobayashi 2015). N1 cells show elevated expression of immunoactive cytokines and chemokines, a lower level of arginase, and a wider capacity for killing neoplastic cells in vitro (Pekarek et al. 1995; Shen et al. 2007). N1 neutrophils promote the recruitment and activation of CD8^+^ T cells through the production of CCL-3, CXCL9, CXCL10 and proinflammatory cytokines such as IL-12, TNF-α and GM-CSF (Fridlender and Albelda 2012). It was also found that the neutrophil–CD8^+^ interaction is crucial in the course of the anticancer immune response, as the lack of neutrophils in the reaction site effectively reduces the response by CD8^+^ T cells (Suttmann et al. 2006).

Pro-neoplastic properties of the N2 phenotype cells are associated with the production of proangiogenic and growth factors (e.g., VEGF: vascular endothelial growth factor), as well as enzymes degrading the extracellular matrix (e.g., MMP-9: matrix metalloproteinase 9). It is also believed that TANs/N2 are capable of inhibiting the immune response against cancer cells by influencing the activity of other cells (Jabłońska et al. 2012; Schmielau and Finn 2001; Tazawa et al. 2003).

Currently attempts are being made at determining the factors that influence the differentiation of cells into N1 and N2 populations. It was established that transforming growth factor (TGF)-β is important in switching the TANs phenotype. Fridlender et al. (2009) indicated that blocking TGF-β significantly slows down the growth of the neoplastic process through the activation of CD8^+^ T cells and macrophages. Moreover, the inactivity of TGF-β results in increased amounts of chemokines for PMNs and, consequently, the recruitment of murine CD11b^+^/Ly6G^+^ TANs with hypersegmented nuclei, elevated cytotoxicity, and high expression of proinflammatory cytokines. The results of these studies indicate that the presence of TGF-β in the neoplasm microenvironment induced the activity of N2 TANs population with a pro-neoplastic phenotype (Fridlender et al. 2009).

According to other authors, the switch between N1 and N2 TANs phenotype is connected with advancement stage of the disease. Their findings indicated that TANs isolated from the lungs of patients with early-stage cancer do not demonstrate immunosuppressive properties but rather stimulate the immune response (Eruslanov et al. 2014).

## Proangiogenic Neutrophil Populations in Response to Transplants

Despite numerous evidence confirming the existence of neutrophil subpopulations, the opponents of the thesis suggest that neutrophils are highly “flexible” cells which allow them to adapt to the environment. The results obtained by Christoffersson et al. (2012) prove that there are two distinct populations of two distinct populations before moving to the tissue, in place of the immune response. The authors discovered a proangiogenic population of PMNs with a high level of CXCR4, recruited at the transplantation location by VEGF-A and releasing MMP-9, enabling the revascularization of the transplanted tissue. The use of VEGF-A or macrophage inflammatory protein 2 to induce this phenotype of PMNs did not produce the expected result, suggesting that this subpopulation of two distinct populations exists even before the contact with the reaction site (Christoffersson et al. 2012).

## Conclusion

This article is to draw the reader’s attention to the difficulty in assessing the heterogeneity of PMNs. The authors of different studies suggest the variable role of neutrophils in depending on conditions. Therefore, it is difficult to determine the classic phenotype of PMNs on the basis of current knowledge about these cells.

Based on the above information, it may well be assumed that the wide scope of activity attributed to neutrophils is connected with the existence of different subpopulations of these cells. Perhaps the pleiotropic activity of neutrophils is connected with the level of differentiation, which largely depends on the cytokine microenvironment and the current need for specific phenotype cells, and therefore, a specific function. Research conducted to identify neutrophil subpopulations must be continued as more and more questions arise about the multitude of functions of these cells. Exploring knowledge about the different, and sometimes even contradictory, aspects of neutrophil biology once be used as a therapeutic instrument in the treatment of disorders which involve neutrophils.
